# Precision medicine in the era of artificial intelligence: implications in chronic disease management

**DOI:** 10.1186/s12967-020-02658-5

**Published:** 2020-12-09

**Authors:** Murugan Subramanian, Anne Wojtusciszyn, Lucie Favre, Sabri Boughorbel, Jingxuan Shan, Khaled B. Letaief, Nelly Pitteloud, Lotfi Chouchane

**Affiliations:** 1 Department of Microbiology and Immunology, Weill Cornell Medicine, New York, USA; 2 Genetic Intelligence Laboratory, Weill Cornell Medicine-Qatar, Qatar Foundation, Doha, Qatar; 3 Service of Endocrinology, Diabetology and Metabolism, Lausanne University Hospital, Lausanne, Switzerland; 4 Clinical Bioinformatics Section, Research Division, Sidra Medicine, Doha, Qatar; 5 Department of Genetic Medicine, Weill Cornell Medicine, 45 E 69th Street, Suite 432, New York, NY 10021 USA; 6 Department of Electronic and Computer Engineering, Hong Kong University of Science and Technology, Kowloon, Hong Kong

**Keywords:** Exposome, Chronic inflammation, Chronic diseases, Precision medicine, Personalized treatment, Deep phenotyping, Big-data analytics, Machine leaning, Artificial intelligence

## Abstract

Aberrant metabolism is the root cause of several serious health issues, creating a huge burden to health and leading to diminished life expectancy. A dysregulated metabolism induces the secretion of several molecules which in turn trigger the inflammatory pathway. Inflammation is the natural reaction of the immune system to a variety of stimuli, such as pathogens, damaged cells, and harmful substances. Metabolically triggered inflammation, also called metaflammation or low-grade chronic inflammation, is the consequence of a synergic interaction between the host and the exposome—a combination of environmental drivers, including diet, lifestyle, pollutants and other factors throughout the life span of an individual. Various levels of chronic inflammation are associated with several lifestyle-related diseases such as diabetes, obesity, metabolic associated fatty liver disease (MAFLD), cancers, cardiovascular disorders (CVDs), autoimmune diseases, and chronic lung diseases. Chronic diseases are a growing concern worldwide, placing a heavy burden on individuals, families, governments, and health-care systems. New strategies are needed to empower communities worldwide to prevent and treat these diseases. Precision medicine provides a model for the next generation of lifestyle modification. This will capitalize on the dynamic interaction between an individual’s biology, lifestyle, behavior, and environment. The aim of precision medicine is to design and improve diagnosis, therapeutics and prognostication through the use of large complex datasets that incorporate individual gene, function, and environmental variations. The implementation of high-performance computing (HPC) and artificial intelligence (AI) can predict risks with greater accuracy based on available multidimensional clinical and biological datasets. AI-powered precision medicine provides clinicians with an opportunity to specifically tailor early interventions to each individual. In this article, we discuss the strengths and limitations of existing and evolving recent, data-driven technologies, such as AI, in preventing, treating and reversing lifestyle-related diseases.

## Introduction

The average lifespan of humans has more than doubled in the last two hundred years, largely due to modern medicine and public health initiatives. However, an extended lifespan is associated with increases in various types of diseases among which noncommunicable diseases (NCDs), also commonly referred to as chronic diseases. Recent evidence indicates that chronic inflammatory diseases are the most significant cause of death worldwide, with over 50% of all deaths due to inflammatory conditions. For this review, we collectively refer to the following as chronic diseases: type 2 diabetes, obesity, cardiovascular disease (CVD), metabolic associated fatty liver disease (MAFLD), cancer, chronic lung and kidney disease, autoimmune and neurodegenerative diseases [1]. Today, our genes function in a world that is completely different from the one they were designed for and modern humans are subjected to an environment that has changed tremendously over the past century. The genetic predisposition to various diseases differs, from person to person and non-genetic factors pose high attributable risks, often assessed between 80 and 90% of the total risk [2, 3]. The Global Burden of Disease (GBD) study, which measured the disease burden of behavioral, environmental and occupational, and metabolic risks or clusters of risks from 1990 to 2016 in 195 countries concluded that the modifiable risk factors lead to nearly 60% of deaths worldwide [4]. Lifestyle-associated chronic diseases tend to have two common characteristics: one is homeostasis disturbance and the second is metaflammation or chronic metabolic inflammation. Therefore, the pathophysiology of chronic diseases points to the physiological rationale that connects inflammation with homeostasis [5]. It is now widely recognized that pathogenesis of disease is often the result of interactions between various genetic and environmental factors. The sum of environmental exposures (non-genetic) from conception until old age, throughout the lifespan is known as the “exposome”. The term "exposome" is used to demonstrate the complexity and extent of exposures to toxic substances, nutrition, psychosocial stressors and physical impacts and their associated biological responses. Exposomics is the study of the exposome, based on the use of internal and external assessment methods [3, 6].

Precision medicine is an emerging field in therapeutics based on an understanding of the genetic make-up, personal lifestyle, gene, and surrounding environment of an individual. We can use precision medicine to customize prevention and treatment strategies for an individual by identifying the factors that predispose this individual to a specific disease and defining the underlying molecular mechanisms that induce the disorder. The use of “OMICS” or “EXPOsOMICS” along with wearable sensors as measurement/assessment methods have the potential to generate large amounts of data (big-data), thus requiring new digital approaches and resources for analyzing, integrating, and interpreting the massive amounts of data [7, 8]. Artificial intelligence (AI): an emerging field in which computer algorithms are equipped to carry out tasks independently of human guidance. To create an efficient AI algorithm, computer systems are initially fed data that is usually organized, indicating each data point has an algorithm-recognizable label or annotation. After sufficient sets of data points and their labels are presented to the algorithm, output is evaluated to ensure accuracy. Such AI algorithms are capable of observing, analyzing vast data and identifying patterns with incredible efficiency [9]. Artificial Intelligence that we consider in this context includes machine learning (ML), deep learning (DL), and artificial neural networks (ANN). When AI is combined with high performance computing approaches, AI allows us to establish and predict disease risk based on individual’s data [10]. Translating such enormous data into clinical knowledge is now in the hands of ML/AI platforms. Promising results in predicting disease risk with greater accuracy have been shown on these platforms [11–14]. As AI enters the world of precision medicine, it can help organizations to capitalize on precision medicine in many ways and help deepen our knowledge of the origins and course of chronic diseases.

This review article discusses the potential contribution of lifestyle factors and biological factors -genetic, epigenetics and the microbiome to the development and progression of chronic inflammation. We will also highlight the recent findings on the implementation of ML/AI algorithms in personalized medicine to better manage and prevent chronic diseases.

## Inflammation—a natural response

**Fig. 1:**
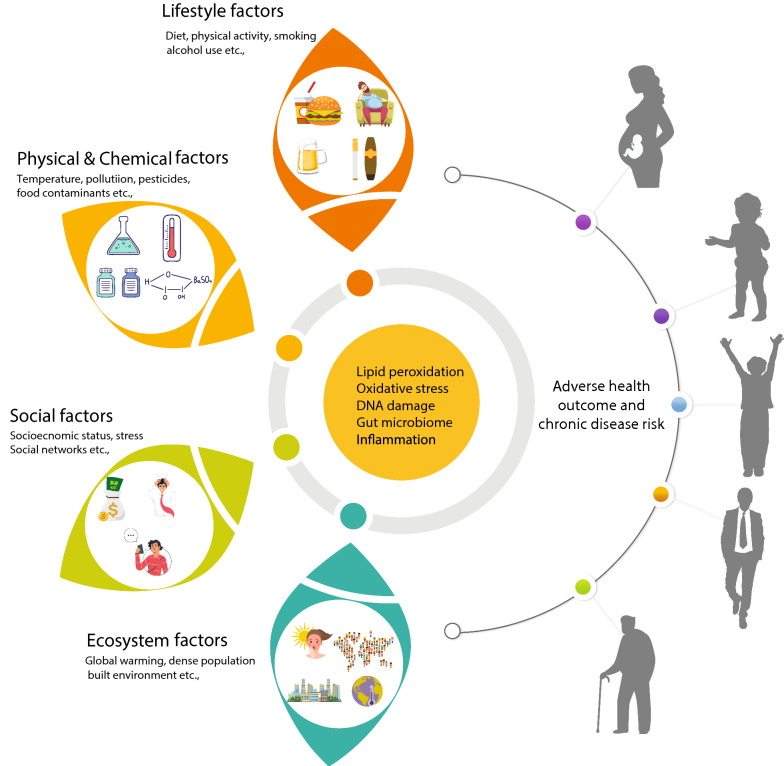
Exposome—internal factors and external environmental factors role in health and disease. The totality of exposure from conception throughout the life course leads to multiple physiological changes in every individual. Internal exposures such as lipid peroxidation, oxidative stress, DNA damage, alterations in gut microbiome, and inflammation collectively plays a major role in health and chronic diseases

In recent years, there has been a substantial improvement in our understanding of the inflammatory mechanism and its contribution to health and diseases. Inflammation is the natural response of the body to harmful pathogens and stimuli in an effort to eliminate threat and/or repair damaged tissue [5]. However, in the early 1990′s a different type of inflammation was associated with overweight and obesity was identified as a persistent and maladaptive inflammatory response that had significant variations compared to classical inflammation [15]. Such systemic inflammation characterized as ‘low-grade’ was associated with elevated levels of inflammatory mediators and increased immune cell infiltration in peripheral tissues without altering the primary function of the tissue [16, 17]. Human exposome can be categorized into external and internal. An increasing number of investigations have been addressing the human exposome, and the external exposome factors were well described in the recent articles [3, 6]. These factors were classified into four categories: (1) Lifestyle factors, such as diet, physical activity, sleep, smoking and alcohol; (2) Physical and chemical factors, such as temperature, pollution, pesticides, food contaminants etc.; (3) Ecosystem factors, such as food systems, climate, global warming, built environment, dense population etc.; (4) Social factors, such as socioeconomic status, stress, social networks, cultural standards etc. [3]. (Fig. 1). An example of environmental chemicals inducing inflammation was shown in a recent study: chemicals such as linuron (an herbicide used in agriculture) and methyl carbamate (a compound used in the fabric, polymer, and pharmaceutical industries), were shown to enhance astrocyte inflammation and neurological inflammation [18]. Accumulating evidences linked air pollution to inflammation and to further number of chronic diseases [19–21]. Likewise, the built environment is linked to the dynamics of infectious diseases such as SARS CoV2, especially in contact-borne diseases (aerosols or droplets), and climate change to vector-borne diseases [22, 23]. The internal exposome that includes, (1) molecules generated endogenously from metabolic reactions, such as oxidative stress and lipid peroxidation, (2) infections, (3) gut microbiome, and (4) other natural reactions that affects DNA and proteins within the body (Fig. 1). In addition, social stress, phycological stress, and socioeconomic status were all linked to inflammation and disease risk [24–26]. Over the past decades, extensive efforts have been made and continue to be made in pursuit of identifying the risk factors for chronic diseases [27–29]. The etiology of chronic diseases has now been convincingly linked to systemic chronic inflammation (SCI). An overwhelming body of evidences and a recent critical review have highlighted the importance of SCI and its correlation with health and chronic diseases [30].

## Lifestyle factors: chronic inflammation axis

The April 2002 issue of Science discussed “The puzzle of complex diseases” where diet and lifestyle were identified to be important contributors for major chronic diseases [31, 32]. Since then, numerous landmark epidemiological and biological studies have recognized that lifestyle related choices and behaviors have contributed significantly to the incidence of chronic diseases. Several studies have linked being overweight and obese with increased risk of chronic diseases, cancers, including breast cancer (in post-menopausal women), endometrium, esophagus, pancreas, liver, colorectum and kidney, and others [33, 34]. A principal lifestyle factor influencing the onset of such diseases is the diet and nutrition of each individual [35, 36]. Evidences from preclinical investigations as well as observational and interventional trials indicated that Western-type diet (WD) is a key driver of chronic, low-grade metabolic inflammation [37, 38]. The consumption of calorie-rich foods: highly processed, ultra-processed (formulations of many ingredients), in addition to sugar sweetened beverages, fructose-containing sugars, trans fats and saturated fats, salt, and other food additives have been proven to influence inflammation and lead to chronic diseases [39–43]. In addition, WD and other popular diets have been shown to alter intestinal microbiome, which in turn helps shape integrated immune responses. Prolonged consumption of such diets leads to disruption of the gut-barrier integrity, allowing harmful secretion of microbial products that can cause inflammation [44–47]. A recent study indicated that the consumption of high-fat ketogenic diets can alter the microbiome and also reduce the number of intestinal pro-inflammatory Th17 cells which are critical for acting against infectious disease [46]. The consumption of fructose-rich diet has emerged as a major contributor to dyslipidemia, NALFD, insulin resistance, and diabetes. A recent elegant study has revealed that intestinal microbiota plays a major role in converting the dietary fructose to acetate, which in turn activates the hepatic lipogenesis [48, 49]. Numerous other dietary factors have also been shown to induce inflammation and lead to SCI. These include the consumption of highly processed foods that lack essential vitamins and micronutrients and the deficiencies in minerals such as calcium, phosphorus, zinc, and magnesium [50–54]. Noteworthy, the recent Lancet Commission report on the global syndemic investigated how the human-driven methods of food production, food supply, consumption and its consequences impacting the environment and climate change. In order to counter the global syndemic of obesity, undernutrition and climate change the commission is urging the governments to reconsider the food supply chains, and business models [55].

Other lifestyle variables such as physical inactivity, lack of sleep, and tobacco smoking, can also activate multiple immune-inflammatory pathways leading to chronic inflammatory conditions [56]. Inadequate physical activity is a leading risk factor for chronic diseases and mortality. Globally, the age-standardized prevalence of insufficient physical activity was 27.5% in 2016 [57]. In addition, over 80% of the world's adolescents are not physically active enough. Several studies have linked insufficient physical activity with inflammatory conditions; even moderate physical activity has demonstrated to possess anti-inflammatory effects, further reducing the risk of chronic diseases and improving age-related multi-morbidity by strengthening the immune function [58, 59]. Tobacco-related morbidity and mortality is significant worldwide since smoking increases the risk of developing a number of serious inflammatory conditions [60]. It has been well established that the nicotine present in the tobacco stimulates neutrophils, with subsequent release of certain molecules that promote inflammation increasing in turn the risk of chronic diseases [61, 62]. Excessive alcohol use damages not only gut and liver functions, but also multi-organ interactions, contributing to chronic inflammation and eventually, increasing the risk of chronic liver diseases and certain cancers [63]. Accumulating evidence suggests that the pathogenesis of psychological disorders, such as depression and anxiety, are also associated with chronic stress and neuroinflammation [64]. Given the clear linkage of lifestyle factors with chronic diseases, their prominence in preventing diseases cannot be over-emphasized.

## Biological factors: genetics and epigenetics

During recent years, there has been tremendous interest in the discovery of genes that are responsible for chronic diseases. Genetic variation consists of differences in the DNA sequences of individuals manifesting as single nucleotide polymorphisms (SNPs), insertions and deletions, and other structural variations. Genome-wide association studies (GWAS) that include analyses of genetic variants across several human genomes in order to detect associations between genotype and phenotype. These have facilitated a remarkable range of discoveries in the biology of chronic diseases [65]. Several studies have found new genetic loci and genes that predispose an individual to a number of chronic diseases, such as type 1 and type 2 diabetes, coronary heart disease, obesity, asthma, cancer, bipolar disorder, depression, rheumatoid arthritis, Crohn's disease, and hypertension [66–73]. In addition, a recent large-scale GWAS conducted among the Japanese population has identified 320 independent signals in 276 genetic loci for 27 diseases among which 25 novel loci, including certain loci specific to, the Japanese population [74]. Due to the diversity in genetic make-up and associated disease variants across populations, data obtained from one population may not be applicable to other populations [75]. However, understanding the variants, genes and mechanisms involved in specific diseases unlocks the possibilities for innovative treatments, diagnostic approaches and the efficient prevention of diseases. Candidate gene and GWAS studies have identified numerous SNPs-genetic susceptibility loci across human genome which explain only a fraction of the inter-individual variation for chronic diseases. To date, however, it has not been shown that solely defined genetic influences contribute to a large proportion of chronic disease incidence at population level.

Beyond lifestyle factors and genetic susceptibility, another powerful determinant of the health outcome is epigenetics. Epigenetic alterations have emerged as surrogate markers for environmental exposure. Recently, epigenetic mechanisms have been increasingly recognized as a critical link between environmental exposure and disease risk [76]. Evidence indicates that maternally regulated environmental modulation of gene expression in offspring and gene-environment interactions are significant determinants of disease risk in later life [77, 78]. In addition, using a unique cohort of more than 700 pairs of monozygotic and dizygotic twins, it was demonstrated that both genetics and environment-inherited epigenetic signatures plays major role in regulating gene expression in the offspring [79, 80]. Moreover, changes in epigenetics are the core mechanisms by which early nutritional conditions can increase later-life susceptibility to obesity and other chronic diseases [81]. Maternal malnutrition influences altered epigenetic regulation in genes that control the metabolism of lipids and carbohydrates and those involved in the neural networks of central appetite-energy homeostasis [82]. This suggests that early experience may lead to changes in the epigenome influencing metabolic and physiological pathways, possibly changing individual’s phenotypic development and thus having critical effect on their health. As stated above, several studies have indicated that dietary components induces alterations in the genome and have linked SNPs interactions with the consumption of particular food and dietary patterns [83–85]. Nutrients and other environmental factors, either directly or indirectly, can impact the levels and turnover of epigenetic signatures (DNA methylation, acetylation of histones) thereby regulating the expression of messenger RNAs and non-coding RNAs that have been implicated in multiple chronic diseases. In summary, epigenetic mechanisms have been shown to be associated with multiple lifestyle factors or environmental exposures, including overnutrition, undernutrition, physical activity, stress, pollutants, and obesity, which have in turn been linked to chronic diseases [86–89].

## Gut microbiome

Multiple studies have discovered that the microbiome impacts almost every aspect of human health, and that the microbial composition, which differs from individual to individual, can be a key component in diverse manifestations ranging from gaining weight to developing stress and depression [90]. Some studies on human or mice microbiome have indicated that this variability begins with variations in host genetics [91, 92]. Several other parallel studies have found that the environment is dominant over host genetics in the development of human intestinal microbiota [93, 94]. A recent study, which looked into factors that influence the intestinal microbiome composition across nine different primate species and four human communities subject to various subsistence habit, identified environmental factors as the main driver of intestinal microbiome composition when compared to host species phylogenies [95]. In addition, the intestinal bacteria in four Himalayan populations (Tharu, Raute, Raji and the Chepang) differed according to their dietary lifestyles [96]. Such findings show that diet can dominate phylogenetic development of gut microbiome composition. Acute dietary changes (four days) was sufficient enough to bring about significant alteration to the human gut microbiota composition [97]. In a recent study that investigated on Irish traveler’s intestinal microbiome shown that microbiota is considerably different from that of a non-traveler settled population. However, the non-travelers (settled) Irish contain microbiota similar to people lives in industrialized society with a comparatively higher risk of chronic disease. Most travelers contain an ancient of microbiome that protects themselves from various chronic inflammatory conditions [98]. Moreover, microbiota-accessible carbohydrates (MACs) serve as an energy source for gut bacteria, resulting in the production of short chain fatty acids (SCFAs) which benefits the host. Further, these SCFAs including butyrate and propionate have multiple effects on signaling pathways including energy homeostasis, carbohydrate and lipid metabolism, and inhibition of inflammatory signals [99, 100].

Moreover, evidence has shown that microbial colonization of the infant occurs at birth through the vaginal canal and also some by breastfeeding and skin-to-skin contact. Babies delivered by caesarean section lack some strains of gut bacteria [101]. Furthermore, environmental exposure early in life has a strong effect on a child’s intestinal microbiome, and studies have linked environmental factors during infancy with a subsequent risk of developing allergies and asthma [102]

## Precision medicine

In the last decade, strategies to advance precision medicine have attracted considerable investment in developing new treatments, understanding more about disease mechanisms, and eventually preventing disease. Precision medicine focused on identifying the effective approaches and the tailored treatment based on an individual’s genetic, environmental, and lifestyle factors. As explained above, we have undeniable evidence of human biological diversity in both health and disease, as shown by the findings of the Human Functional Genomics Project (HFGP) focused on 500 healthy adult subjects [103]. A number of studies have illustrated this explicitly by analyzing immune cells (cytokine) as an endpoint, showing that the cytokine types and levels vary depending on environmental factors (e.g., season driven), genetic history, and intestinal microbiome composition [104]. Furthermore, the latest study from the HFGP has shown that 11 different kinds of host factors together accounted for up to 67% of inter-individual variation in activated cytokine production in healthy subjects [105].

**Fig. 2:**
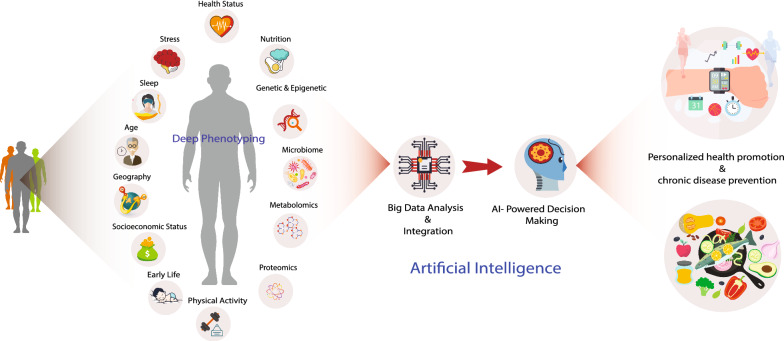
Deep phenotyping and artificial intelligence for health promotion and chronic disease prevention. Deep phenotyping provides an entire molecular profile of an individual’s physiological status. When longitudinally tested, the pathways can be tracked to identify the transformation from a health to a disease. Various omics technologies along with other physiological measurements will be used to molecularly characterize an individual’s risk for disease. Further implementation of a systems approach to the big-data analysis and integration will provide a platform for machine learning and artificial intelligence in clinical decision-making for early disease risk identification and prevention

Overall, interpersonal variability in diet, lifestyle, sleep, stress, socioeconomic status, geography, early life experiences and exercise habits combined with gut microbiome, genetic background, metabolism, inflammatory status, are all critical factors in determining an individual’s heath and risk for disease (Fig. 2). In addition, the exposure of individual to environmental hazards is not constant and can change throughout their life, and also the effect of the exposures can vary depending on an individual's life stage. Environment-wide association studies (EWAS) have been proposed to examine new environmental factors in disease risk [106]. Therefore, the need of every individual is complex and require in-depth assessment (deep phenotyping) before interventions can be confidently applied (Fig. 2).

## Deep phenotyping and artificial intelligence

Precision oncology, AI can be used to develop a drug combination centered on a patient's own biopsy and adopt N-of-1 medication recommendations [107]. Across multiple specializations, especially in radiology AI-based algorithms have already shown improvement in diagnostic accuracy and performance [108–111]. The US Food and Drug Administration (FDA) has licensed many AI systems to promote medical imaging evaluation, including the detection of abnormal lesions that may progress to cancer [112]. A recent work highlights how AI and the advancement of technologies together are empowering the aim of personalized and precision medicine [113].

Machine learning is a key for multi omics data integration and there are several aspects in which data types are combined and their relationships are explored [114]. One of the large prospective cohort studies the UK Biobank project has collected deep genetic and phenotypic data including biological measurements, lifestyle markers, blood and urine biomarkers, and brain imaging from 500,000 individuals. This project has provided researchers with opportunities to search for genetic associations with disease risk and has resulted in several publications [115]. In addition, a precision medicine screening study that introduced a platform of deep quantitative multimodal phenotyping including genomics, metagenomics, advanced imaging, metabolomics, clinical testing and family history, provided a comprehensive, predictive, and personalized assessment of individuals health and chronic disease risk [116].

Further to big data obtained from deep phenotyping, a recent study empowered its participants with an additional behavioral coach. The Pioneer 100 wellness project (P100) was an initial effort to obtain and analyze large omics data sets to correlate molecular networks in 108 healthy individual. This study performed whole genome sequencing, proteome, microbiome, metabolome, recorded clinical data, daily physical activity, and sleep patterns for every three months over a nine months period. The investigators established personal, dense, dynamic data clouds for each participant and carried out an integrated analysis of six different data types. Further, these data-driven insights combined with behavioral coaching significantly improved the wellness of the participants with regard to nutrition, inflammation, diabetes and CVDs [117]. A similar study performed deep longitudinal omics profiling along with wearable monitoring for 109 individuals who were at increased risk for diabetes. They utilized multi-omics including genome, transcriptome, immunome, metabolome, proteome, and gut microbiome measured for up to eight years (median, 2.8 years). Such a deep analysis for this long period of time allowed the recognition of 67 clinically actionable health outcomes, including the cardiovascular disease risk [118]. Furthermore, in order to understand the molecular changes of the ageing process and associated disease risk, a recent study performed longitudinal and deep multi-omics profiling from 106 healthy individuals aged between 29 to 75 years and analyzed how diverse types of ‘omics’ results combined with clinical markers, correlated with age. This study identified various types of aging patterns called 'ageotypes,' based on the types of molecular pathways that have evolved over time in a specific person. Such ageotypes provides new possibilities for the design of early diagnosis and treatment interventions that may slow down the aging process depending on the particular biology of each individual [119].

Machine learning has been widely applied to the precision nutrition field to customize a personalized diet aimed to prevent or manage diet related diseases [120, 121]. One landmark study has successfully used a precision nutrition approach and has created a personalized diet to predict blood glucose response by considering biochemical, anthropometrics, dietary intake, physical activity, and gut microbiota data in an integrated framework. In this study, 800 healthy and prediabetic individuals were examined and their responses to the food were measured a total of 46,898 meals. The investigators adopted an ML/AI algorithm that precisely predicted postprandial glycemic responses (PPGRs) to meals. The ML/AI predictions were validated in an independent 100-individual cohort. Finally, a blinded, randomized controlled intervention based on an algorithm predicted diet resulted in significantly lower PPGRs and consistent changes in gut microbiota composition [122]. Recent independent similar studies using the personalized nutrition strategy for PPGRs to diet was confirmed in healthy individuals in an American population [123, 124]. Moreover, ML/AI is transforming the electronic health record (EHR) field and over time EHRs powered with AI were shown to reveal more about diseases. The ML/AI, tools applied to the health records of patients in EHRs and accurately predicted their probability of acquiring or developing chronic diseases [125].

## AI medical assistants

The management of chronic disease requires regular monitoring and recommendations. Virtual medical assistants using AI have recently matured and are being used in various products. AI assistants for diabetes have been shown useful to control patient conditions. For example, Onduo is a company that provides a virtual coaching via text messages through a mobile app. It uses AI technology for food recognition, glucose sensor and physical activities to provide recommendations. Other examples of startup companies are Virta, Wellpepper or Accolade. Another interesting solution is provided by DayTwo. It gives a personalized nutrition recommendation based on subject’s gut microbiome. The suggested meals are chosen among a large database of more than 100,000 foods to keep the glycemic range under control [123].

For cardiac diseases, AI has shown major progress in the diagnosis of atrial fibrillation. The latter is a common problem represents a 20 to 30% lifetime risk. It can occur without symptoms and increase the risk of stroke. AliveCor developed a system based on deep learning, single-lead ECG sensor and physical activity via accelerometer data [126]. The system is integrated with a smart watch and it is capable of predicting the occurrence of atrial fibrillation every 5 s. AI has also contributed in improving diagnoses based on cardiovascular imaging such as in echocardiography, MRI or ultrasound imaging. A comparative study of echocardiography interpretation of Ultromics system with cardiologist showed that more than 90 percent of the abnormalities found by board certified cardiologists overlapped with the ones found by the AI system [127].

Another example of the use of AI for chronic disease management is ResApp Health. The system uses the phone microphone to analyze the subject’s breathing. The AI algorithm is able to give an assessment of several lung conditions with high accuracy such as chronic obstructive lung disease, pneumonia or chronic asthma [128].

Taken together, while high-throughput data generation strategies are becoming more advanced, quicker, and comparatively more affordable, researchers are increasingly gaining access to large amounts of molecular knowledge from human cohorts. The volume of potential data is enormous, and it has been estimated that personal lifestyle-based data sum up to 1100 terabytes over a lifespan, with genetics and clinical data comprising 6.4 terabytes, which is less than 1% of the total. Omics technologies, GWAS, EWAS, smartphone-based digital phenotyping, sensors, EHRs, wearable devices to monitor physical activity, geography location data and climate data combined with AI have improved the prospect of implementing prevention and management strategies for chronic diseases [129–132]. Therefore, the use of such large multidimensional data requires the establishment of structured collection and big data analytics, as well as multidisciplinary integration of high-performance computational technologies and integration of ML/AI. Hence, AI is quickly becoming a crucial methodology in the advancement of precision/personalized medicine [133, 134].

## Recent developments in the field

One of the first large-scale, population-based, prospective studies which intended to enable comprehensive analyses of the genetic and non-genetic causes of diseases for middle and old age was the UK biobank study [115, 135]. In addition, the most ambitious longitudinal study in precision medicine so far, the “All of Us” Research Program, which aims to focus research on the link between environment, lifestyle and biology in health and disease is ongoing [136]. The All of Us program intends to enroll one million people across America and plans to implement deep phenotyping by gathering genetic and health data (using EHRs, digital health data), geography, and biospecimens for biomarker review. Similarly, a project called “The Project Baseline Health Study (PBHS)” was initiated to map human health by deep phenotyping to at least 10,000 individuals. The PBHS study established a portal that incorporates and analyzes personalized, longitudinal, multidimensional data, with a greater focus on future than past. It further explore the biological heterogeneity of healthy individuals or individuals with chronic disease in detail for a longer period of time to create reference health status by integrating various aspects of health [137].

The Human Exposome Project, 2020 from the European Union is the largest network of research programs aiming to address the environmental exposure such as diet, lifestyle, occupational and other environmental factors impact on human health (https://www.humanexposome.eu/). Such data-driven approach to exposome reduces the conventional decision-making method and it may better determine the influence of chemical exposures on particular physiological systems proven to be affected. Subsequently this would help to create novel chemicals with reduced impact on human health and the environment [138]. Over the last two decades, omics, wearables, sensors, digital medicine and emerging innovative technologies together with AI have all made incredible advancements in the field of precision/personalized medicine. Furthermore, AI is being implemented in precision oncology to help clinicians in decision making, with the aim of improving patient outcomes [139]. AI-based healthcare practices are already being implemented in high-income countries; for instance, the UK and Singapore have recently launched national strategies to tackle chronic disease burden using AI. Data driven, AI-powered health care has the potential to clarify the landscape of findings and enable clinical decisions to digitally identify, treat, and manage chronic conditions.

## Data protection and privacy

The frequent collection of personal health and environmental data has been greatly improved through the use of decentralized sensors, measurement devices and mobile phones. A few decades ago, the measurement of blood pressure, glucose level, heart rate could only be done by medical experts. Nowadays such information can be continuously collected through mobile apps. The rapid introduction of AI technology into the precision medicine is advantageous, as AI offers an opportunity to increase the efficiency of health care delivery and the quality of patient care. However, it is necessary to mitigate the ethical risks of the AI implementation, which could include data privacy and confidentiality violations, informed consent, and patient autonomy. In the world of precision medicine, big data and AI, it is of paramount importance that data protection legislation is in place that properly ensures the privacy of individuals, particularly patients. The raise of privacy concerns related to the collection of health data has contributed in the significant progress on private AI methods such as Federated Learning or Differential Privacy in Machine Learning [140]. Countries around the world introducing laws to protect the privacy of their citizens. The Health Insurance Portability and Accountability Act (HIPAA) in USA the primary federal law to protect the privacy of health data. However, HIPAA has major gaps in current world because it protects only relevant health information produced by "covered entities" or their "business associates” [141, 142]. Whereas in Europe, The General Data Protection Regulation (GDPR) has been practical since May 25, 2018 in all European Union (EU) member states and implemented a new era of extensive data protection law within the EU [143, 144]. GDPR regulation has begun a significant global shift in data protection, creating political campaigns that advocate more privacy for data subjects, stricter laws for private corporations and governments that control emerging and increasingly evolving technology that pose a threat to data security.

## Conclusion

Chronic diseases impose a substantial health and economic burden worldwide, with nearly one in four adults suffering from one or more chronic health conditions. To date, the longitudinal cohort studies have set the stage for enhancing human health by identifying and defining the natural history of diseases, identifying their risk factors and finding novel biomarkers. Further, the use of biosensors and the advances in multi-omics have established the foundation for better disease categorization, created targeted therapies, and have improved prognosis for many diseases. Most importantly, advances in digital medicine have helped to determine the underlying causes of diseases in individual patients.

Since most chronic diseases are the consequence of primary lifestyle factors, individuals can reduce the likelihood of developing chronic conditions by making healthier lifestyle decisions. Nutrition and lifestyle preferences are affected by a wide variety of socio-economic factors including employment, education, geography, built environment, social networks, and a climate system. Combatting obesity and chronic diseases associated with diet needs careful examination of the social determinants of food systems, environment and climate change and specific public health strategies targeted at minimizing health disparities [55, 145, 146].

One aim of public health is promoting healthy lifestyle and developing novel approaches to prevent, detect, and respond to diseases that commonly affect people. With the development of precision medicine and the advent of AI, it can be misconstrued that medicine and health care is again drifting towards an individualistic approach versus a community approach to controlling diseases [147]. On the contrary, precision medicine, AI, and our deep understanding of disease conditions offer a great opportunity to save resources for those countries that have practiced a one-size-fits all and a piecemeal approach in their public health thinking and programming and have not reaped adequate return for their investments. Chronic diseases, and their multifactorial nature, the advent of technological advancements in the form of AI, and the ‘precision’ in precision medicine have the potential to redefine and replace conventional public health approaches with a new holistic paradigm [148]. There remains a huge scope for introducing educational programmes, developing policies, and strengthening systems to capitalize on the rapid development in the field and customize activities for collectives (persons who share common traits and characteristics) rather than communities.

## Data Availability

Not applicable.

## Abbreviations

HPC: High-performance computing
AI: Artificial intelligence
ML: Machine learning
DL: Deep learning
ANN: Artificial neural networks
SCI: Systemic chronic inflammation
NCDs: Noncommunicable diseases
MAFLD: Metabolic associated fatty liver disease
CVDs: Cardiovascular disorders
GBD: Global Burden of Disease
WD: Western-type diet
SNPs: Single nucleotide polymorphisms
GWAS: Genome-wide association studies
EWAS: Environment-wide association studies
MACs: Microbiota-accessible carbohydrates
SCFAs: Short chain fatty acids
HFGP: Human Functional Genomics Project
FDA: Food and Drug Administration
PPGRs: Postprandial glycemic responses
HER: Electronic health record
PBHS: Project Baseline Health Study
